# Outcome Indicators on Interprofessional Collaboration Interventions for Elderly

**DOI:** 10.5334/ijic.2017

**Published:** 2016-05-16

**Authors:** Giannoula Tsakitzidis, Olaf Timmermans, Nadine Callewaert, Veronique Verhoeven, Maja Lopez-Hartmann, Steven Truijen, Herman Meulemans, Paul Van Royen

**Affiliations:** Department of Primary and Interdisciplinary Care of the University of Antwerp, Faculty of medicine and Health Sciences, University of Antwerp, Antwerp, Belgium; Department of Nursing and Midwifery Sciences, Centre for Research and Innovation in Care, University of Antwerp, Antwerp, Belgium and HZ University of Applied Sciences, Vlissingen, The Netherlands; Department of Health Sciences, Artesis-Plantijn University College of Antwerp, Antwerp, Belgium; Department of Rehabilitation Sciences and Physiotherapy, Faculty of Medicine and Health Sciences, University of Antwerp, Belgium; Department of Sociology and Research Centre for Longitudinal and Life Course Studies, University of Antwerp, Antwerp, Belgium

**Keywords:** elderly, interprofessional care, quality of care, effect

## Abstract

**Background:**

Geriatric care increasingly needs more multidisciplinary health care services to deliver the necessary complex and continuous care. The aim of this study is to summarize indicators of effective interprofessional outcomes for this population.

**Method:**

A systematic review is performed in the Cochrane Library, Pubmed (Medline), Embase, Cinahl and Psychinfo with a search until June 2014.

**Results:**

Overall, 689 references were identified of which 29 studies met the inclusion criteria. All outcome indicators were summarized in three categories: collaboration, patient level outcome and costs. Seventeen out of 24 outcome indicators within the category of ‘collaboration’ reached significant difference in advantage of the intervention group. On ‘patient outcome level’ only 15 out of 32 outcome parameters met statistical significance. In the category of ‘costs’ only one study reached statistical significance.

**Discussion and conclusion:**

The overall effects of interprofessional interventions for elderly are positive, but based on heterogeneous outcomes. Outcome indicators of interprofessional collaboration for elderly with a significant effect can be summarized in three main categories: ‘collaboration’, patient level’ and ‘costs’. For ‘collaboration’ the outcome indicators are key elements of collaboration, involved disciplines, professional and patient satisfaction and quality of care. On ‘patient level’ the outcome indicators are pain, fall incidence, quality of life, independence for daily life activities, depression and agitated behaviour, transitions, length of stay in hospital, mortality and period of rehabilitation. ‘Costs’ of interprofessional interventions on short- and long-term for elderly need further investigation. When organizing interprofessional collaboration or interprofessional education these outcome indicators can be considered as important topics to be addressed. Overall more research is needed to gain insight in the process of interprofessional collaboration and so to learn to work interprofessionally.

## Introduction

The ageing of the population is expected to be a major driver of increasing demand for long-term care multi-disciplinary services [1,2]. An average of 81% (for Belgium 84%) Europeans prefers to be cared for in their homes either by relatives or by professionals, whereas only 8% (for Belgium 11%) prefers to be cared for in a long-term care institution [3]. Delivery of health care for the ageing population will therefore require more and high levels of inter-disciplinary teamwork or ‘interprofessional collaboration’ [4,5,6]. The extent to which different health care professionals work inter-disciplinary well together affects the quality of the health care that they provide [7,8,9]. Distinctions between the terms multi-disciplinary and inter-disciplinary (or interprofessional) are important. Interprofessional collaboration (IPC) is a model of different disciplines (inter-disciplinary) working together [10,11,12] and assumes a process by which professionals develop an integrated and cohesive answer to the needs of the care receivers and their social system [13,14]. In multi-professional collaboration on the contrary, appropriate experts from different disciplines handle problems of care receivers independently. The care receivers’ problems are subdivided and treated separately, with each provider responsible for his/her own area so it is more an additive collaboration rather than an integrative collaboration as in IPC [15]. Despite the large amount of publications on IPC, still a higher quality of research, evidence and more rigorous evaluation is needed to understand the effectiveness of IPC and to support decision makers [9,16]. Studies should provide insights into how interventions affect collaboration and how improved collaboration contributes to changes in outcomes on patient level and especially quality of care [9]. Over the years different studies tried to indicate positive effects of IPC and interprofessional education (IPE) in practice for outcomes on patients [9,17]. However indicators to measure the effect of IPC in order to learn to collaborate interprofessionally, are still not well investigated nor standardized [18,19]. A summary of outcome indicators used to measure the effect of IPC interventions for elderly, can help to organize IPC and to develop IPE. An overview of effective indicators of IPC can help to gain insight in how interventions affect collaboration and how improved collaboration contributes to changes in outcomes for elderly. This review aims to summarize outcome indicators used to measure the effect of IPC interventions for elderly.

## Methods

### Search strategies

A systematic search was performed for articles published between 2007 and June 2014. This search for relevant publications repeated the strategy used by Zwarenstein et al 2009 [9] as a starting point not with the aim to update the review. Databases used were The Cochrane Library, Pubmed (Medline), Embase, Cinahl and Psychinfo. Only literature published between 2007- and June 2014 was included. The search strategy employed the following terms: interprofessional relations, patient care teams, interprofessional, multidisciplinary and transdisciplinary collaboration strings as used can be found in annex.

### Selection criteria publications

For the search five independent readers (GT, NC, VV and MLH) selected the references on the basis of title and abstract using the following inclusion criteria: a practice-based IPC intervention was the topic of the study and outcomes were reported on the effect of the IPC intervention with a relevance for elderly. We also reviewed the selected studies on description of the intervention and the control group. An IPC was considered when there was a model of working together between different disciplines and with the awareness of the process by which health care professionals developed an integrated and cohesive answer to the needs of the care receivers and their social system, a common vision and purposeful approach and shared responsibility [13,14,20].

### Study quality appraisal

The selected papers were screened on full text by two reviewers (GT and PVR) and assessed with the use of the Dutch Cochrane assessment instruments for evaluation of systematic reviews, for evaluation of RCT’s, cohort studies and qualitative research [21].

### Data extraction

For all included studies the characteristics were reported including year of publication, study design, population, aim, intervention and control, and finally outcome (see Table 2).

**Table 1:** Results Quality Appraisal.

RCT’s Author (y)	Questions (Dutch Cochrane for RCT’s instrument)									TOTAL/9	Quality appraisal: Medium/High

	1	2	3	4	5	6	7	8	9	
Bellantonio, 2008	1	1	0	0	0	0	0	1	1	4	medium
Berggren, 2008	1	1	1	1	1	1	1	1	1	9	high
Berglund, 2013	1	1	0	0	0	1	1	1	1	6	medium
Boult, 2008	1	1	0	0	0	0	0	1	1	4	medium
Boyd, 2009	1	1	0	0	1	1	1	1	1	7	high
Bryant, 2011	1	0	0	0	0	1	1	1	1	5	medium
Chapman, 2007	1	0	0	0	0	0	1	1	1	4	medium
Counsel!, 2007	1	1	1	1	1	1	1	1	1	9	high
Counsell, 2009	1	1	1	0	0	1	1	1	1	7	high
Denneboom, 2007	1	0	0	0	0	1	1	1	1	5	medium
Hogg, 2009	1	1	0	0	1	1	1	1	1	7	high
Markle-Reid, 2010	1	1	0	0	1	1	1	1	1	7	high
Mudge, 2012	0	0	0	0	1	1	1	1	1	5	medium
Phelan, 2007	1	0	0	0	1	1	1	1	1	6	medium
Respect team, 2010	1	1	0	0	1	1	1	1	1	7	high
Ryvicker, 2011	1	0	0	0	1	1	1	1	1	6	medium
Stenvall, 2007a	1	1	0	0	0	0	1	1	1	5	medium
Stenvall, 2007b	1	1	1	1	1	1	1	1	1	9	high
Unutzer, 2008	1	0	0	0	0	1	0	1	1	4	medium
Van Leeuwen, 2009	1	1	0	0	1	1	1	1	1	7	high
Wu, 2010	1	0	0	0	0	1	1	1	1	5	medium
Young, 2007	1	1	0	0	1	1	1	0	1	6	medium
For all questions 1 = yes 0 = no or? Questions: 1. Randomization? 2. Allocation concealment? 3. Patient blinding? 4. Blinding of administrator of treatment? 5. Blinding outcome assessment? 6. Similarity of groups at the start of the study? 7. Descriptions of losses to follow-up/withdrawals? 8. Intention-to-treat analysis? 9.Groups equally provided of care? Note: Publications with a score < 4 were excluded.											

SR Author (y)	Questions (Dutch Cochrane for SR instrument)								TOTAL/ 8	Medium/High

	1	2	3	4	5	6	7	8		
Gates, 2008	1	1	1	1	1	1	1	1	8	high
Handoll, 2009	1	1	1	1	1	1	1	1	8	high
Nazir, 2013	1	1	1	1	1	1	1	1	8	high
Stroke Unit Trialists’, 2007	1	1	1	1	1	1	1	1	8	high
Cameron, 2010	1	1	1	1	1	1	1	1	8	high
For all questions 1 = yes 0 = no or? Questions: 1. Question adequately formulated? 2. Quality of search? 3. Selection procedure? 4. Quality appraisal? 5. Description of data extraction? 6. Description of study baseline characteristics? 7. Clinical and statistical heterogeneity? 8. Statistical pooling? Note: Publications with a score < 4 were excluded.										

Cross sectional study’s Author (y)	Questions (Dutch Cochrane for cohort research)								TOTAL/8	Medium/High

	1	2	3	4	5	6	7	8		
Dedhia P, 2009	1	0	1	1	0	1	0	1	5	medium
For all questions l = yes à = no or? Questions: 1. Comparable groups defined? 2. Can selection bias be excluded? 3. Is the exposure defined and is the method judging exposured? 4. Is the outcome well defined and is the method judging outcome adequate? 5. Is the outcome blind for exposure defined? 6. Is the follow-up period long enough? 7. Can selective loss-to-follow-up be excluded? 8. Are the important confounders of prognostic factors identified and is this being adapted in the design of the research or the analyses? Note: Publications with a score < 4 were excluded.										

Qualitative research Author (y)	Questions (Dutch Cochrane for qualitative research)							TOTAL/ 7	Medium/High

	1	2	3	4	5	6	7	
Rantz, 2013	1	1	1	0	0	0	1	4	medium
For all questions 1 = yes 0 = no Questions: 1. Relevant research question? 2. Adequate method of data collection? 3. Adequate sampling ? 4. Research is controllable? 5. Concrete description of methods used for analysis? 6. Researcher perspective is described? 7. Conclusion fits qualitative research criteria? Note: Publications with a score < 4 were excluded.									

RCT’s= Randomized controlled trials, SR= Systematic review. A score < 4 is low, between 4 and 6 = medium, > 7 = high

**Table 2:** Overview data-extraction included studies.

Reference	Study design	Population	Aim	Intervention and control	Outcome

Chapman, 2007	RCT	118 residing in nursing homes (Aged ≥75)	This study evaluated the effectiveness of advanced illness care teams (AlCTs) for nursing home residents with advanced	AICT advanced illness care teams versus usual care	Descriptive characteristics of the participants (age education, income, MMSE, Global deterioration scale, ADL-Scale, gender, marital status, race or ethnicity) ^*^ pain ^*^ depression ^*^ agitation With: ^*^ Cohen-Mansjield Agitation Inventory (CMAI) to measure agitated behaviors in elderly people ^*^ Faces Legs Activity Cry Consolability (FLACC) Behavioral Pain Scale. ^*^ Cornell Scale for Depression in Dementia (CSDD). ^*^ Pain in Advanced Dementia (PAINAD).
Counsell, 2007	RCT	951 adults 65 years or older	To test the effectiveness of a geriatric care management model on improving the quality of care for low-income seniors in primary care.	Geriatric resources for assessment and care of elders (grace) versus usual care	Main outcome measures: ^*^ medical outcomes: 36-item short-form (SF-36) scales and summary measures (PCS, physical component summary and MCS, mental component summary) ^*^ instrumental and basic activities od daily living (AHEAD-survey), also days in bed due to illness or injury ^*^ patients’ overall satisfaction ^*^ emergency department visits not resulting in hospitalization and hospitalizations. Also: Depression severity with Patient Health Questionnaire Quality of medical care with ACOVE (Assessing Care Of vulnerable Elders)
Denneboom, 2007	RCT	738 Older people (≥ 75 years) on polypharmacy (> five medicines)	To determine which procedure for treatment reviews (case conferences versus written feedback) results in more medication changes, measured at different moments in time. To determine the costs and savings related to such an intervention.	Pharmacists and GPs performed case conferences on prescription-related problems *vs pharmacists provided resuits of a treatment review in GPs as written feedback*.	^*^ number of medication changes (following recommendations with clinical relevance) ^*^ costs and savings associated with the intervention at various times were calculated.
Phelan, 2007	RCT	874 patients aged 75 and older	To assess the effect or a team of geriatrics specialists on the practice style of primary care providers (PCPs) and the functioning of their patients aged 75 and older.	An interdisciplinary team of geriatrics specialists worked with patients and providers to enhance the geriatric focus of care vs usual care	^*^ Practice level outcomes: - careful prescribing, operationalized as avoidance of prescribing high-risk medications (defined for purposes of this study as psychoactive medications); and proactive screening for selected geriatric syndromes (depression, cognitive impairment, falls). - satisfaction with the Senior Resource Team (SRT) ^*^ patient level outcomes - functional status: Arthritis Impact Measurement Scale 2F ShortForm (AIMS2-SF) - new disability in any basic ADLs (bathing, using the toilet, feeding oneself, and walking inside the home), self-rated health, psychological, well-being (assessed using the Mental Health Index-5), and hospitalizations. ^*^ death ascertainment (24-months follow-up)
Stenvall, 2007a	RCT	199 patients with femoral neck fractures aged 70 years or older	To investigate the short- and long-term effects of a multidisciplinary postoperative rehabilitation programme in patients with femoral neck fracture.	Special Intervention program in geriatric ward *versus conventional care in orthopedic ward*	Short- and long-term effects of intervention on: ^*^ activities of daily living ^*^ mobility after hip fracture (walking ability) ^*^ consumption inpatient days after discharge ^*^ mortality
Stenvall, 2007b	RCT	199 patients with with femoral neck fracture aged ≥ 70 years	This study evaluates whether a postoperative multidisciplinary, intervention program, including systematic assessment and treatment of fall risk factors, active prevention, detection, and treatment of postoperative complications, could reduce inpatient falls and fall-related injuries after a femoral neck fracture.	Special intervention program in geriatric ward vs conventional care in orthopedic ward	^*^ postoperative fall incidence rate ^*^ postoperative complications ^*^ postoperative in-hospital stay
Stroke unit, 2007	SR	Involving 6936 patients of which one subgroup age: greater than 75 years and have had a stroke	To assess the effect of stroke unit care compared with alternative forms of care for patients following a stroke.	Organized inpatient (stroke unit) care	^*^ primaryanalysis examined: death, dependency and type requirement for institutional care ^*^ secondary outcome measures included: quality of life, patient and care satisfaction, duration of stay in hospital or institution or both
Young, 2007	RCT	490 older patients (81–90)	To compare the effect of community hospital care on Independence for older people needing rehabilitation with that of general hospital care.	community hospital rehabilitation versus usual care	^*^ primary outcome: independence with Nottingham extended activities of daily living scale (NEADL) ^*^ secondary outcome: independence with Barthel index; for emotional, social and physical health problems the Nottingham health profile, hospital anxiety and depression scale; mortality; discharge destination; 6-months residence status and satisfaction with services.
Bellantanio, 2008	RCT	100 persons with dementia moving into two dementia-specific assisted living facilities > 70Y.	To determine whether a multidisciplinary team intervention minimizes unanticipated transitions from assisted living for persons with dementia.	Four systematic multidisciplinary assessments conducted by a special geriatric team versus usual clinical care consisted of a medical evaluation conducted by the resident’s primary care physician	Permanent relocation from assisted living to a nursing facility, emergency department (ED) visits, hospitalization, and death. ^*^ socio demographic and medical information age, sex, comorbidities, weight ^*^ Cognitive Status 30-item Folstein MMSE ^*^ Functional Status KATZ-ADL index ^*^ Behavioral Symptoms BehaveAD Rating scale
Bergrenn, 2008	RCT	199 patients with femoral neck fracture aged ≥ 70 years	This study evaluates whether a postoperative multidisciplinary, multifactorial fall-prevention program performed by a geriatric team that reduced inpatient falls and injuries had any continuing effect after discharge. The intervention consisted of staff education, systematic assessment and treatment of fall risk factors and vitamin D and calcium supplementation.	Special intervention program in geriatric ward versus conventional care in orthopedic ward	Comparing falls and new fractures between intervention and control. ^*^ basic characteristics during hospitalization, at 4 months and 12 months. ^*^ medical data ^*^ social data ^*^ including morbidity and mortality, the occurrence of falls.* occurrence of falls were registered from the records (obliged to document)
Boult, 2008	(cluster) RCT	904 multimorbid older patients (66-106y)	To assess whether GC can improve the quality of health care for this population, “Guided Care" (GC) was designed to enhance quality care by integrating a registered nurse, intensively trained in chronic care, into primary care practices to work with physicians in providing comprehensive chronic care to 50–60 multimorbid older patients.	Guided Care versus usual care	^*^ Patients ‘heaith and functional status, quality of health care, and satisfaction with health care. ^*^ Patient Assessment of Chronic Illness Care (PACIC) ^*^ Satisfaction with 11 aspects of care. ^*^ The amounts of time spent on five tasks necessary for managing chronically ill patients. ^*^ Whether the physician knows six elements of information. ^*^ Whether four care coordination processes occur. ^*^ Elements of information and care coordination were derived from the Primary Care Assessment Tool (PCAT).
Gates, 2008	SR	Involving 5874 elderly	To evaluate the effectiveness of multifactorial assessment and intervention programmes to prevent falls and injuries among older adults recruited to trials in primary care, community, or emergency care settings.	Fall prevention interventions versus standard care, no fall prevention intervention	^*^ no of fallers ^*^ fall related injuries ^*^ recurrent falls ^*^ admission to hospital attendance at emergency departments ^*^ attendance at doctor’s surgery ^*^ death ^*^ move to institutional care
Unutzer, 2008	RCT	551, 60 years or older patients with major depression, dysthymia or both	To determine the long-term effects on total healthcare costs of the Improving Mood: Promoting Access to Collaborative Treatment (IMPACT) program for late-life depression compared with usual care.	Collaborative care intervention (IMPACT) vs usual care	cost outcome data
Counsell, 2009	RCT	951, low lncome seniors aged 65 or older	To provide, from the healthcare delivery system perspective, a cost analysis of the Geriatric Re-sources for Assessment and Care of Elders (GRACE) intervention, which is effective in improving quality of care and outcomes.	Home-based care management for 2 years versus usual care.	^*^ chronicen preventive care costs ^*^ acute care costs ^*^ total costs in the full sample (^*^ predefined high-risk and low risk groups)
Dedhia, 2009	pre-post design ‘cohort’	422 patients 65y> admitted to the hospitalist services	To study the feasibility and effectiveness of a discharge planning intervention	Intervention period: October-April 2007 1. admission form with geriatric cues 2. facsimile to the primary care 3. inteicisciplinary worksheet to identify barriers to discharge 4. pharmacist-physician collaborative medication reconciliation 5. predischarge planning appointments vs control period January–May2006	Thirty-day readmission and return to emergency department rates and patient satisfaction with discharge. ^*^ Katz ^*^ self-perceived health status ^*^ ED visits ^*^ need for hospital read mission ^*^ patient satisfaction with Coleman’s Care Transition Measures (discharge planning intervention: ^*^ follow-up within 1 week of discharge ^*^ follow-up at 30 days after discharge Effect of the intervention across the three hospital sites)
Handoll, 2009	SR	Involving 2498 elderly	To examine the effects of multidisciplinary rehabilitaion, in either inpatient or ambulatory care settings, for older patients with hip fracture.	Interventions with treatments in a multidisciplinary rehabilitation program (supervised by geriatrician or rehabilitation physician/clinician) versus usual care	Primary outcome: ^*^ ‘poor outcome’ defined as death or deterioration of functional status leading to increased dependency in the community or admission to institutional care. Secondary outcomes: ^*^ Morbidity ^*^ Length of stay in hospital ano hospital readmission ^*^ Carer burden ^*^ Costs
Hogg, 2009	RCT	241 adults 50 and older and considered to be at risk of experiencing adverse health outcomes	To examine whether quality of care (QQC) improves when nurse practitioners and pharmacists work with family physicians in community practice and focus their work on patients who are 50 years of age and older and considered to be at risk of experiencing adverse health outcomes.	Anticipatory and Preventive team care (APT care) from a collaborative multidisciplinary team versus usual care from family physicians	Main outcome measure: ^*^ chronic disease management score secondary outcomes: ^*^ Intermediate clinical outcomes (mean hemoglobine A_1c_ blood pressure). ^*^ Quality of preventive care ^*^ QOL with the SF-36
Van Leeuwen, 2009	Multisite RCT	906 Young-old (60-74y) and old-old patients (≥ 75y)	To compare the clinical outcome of young-old patients and old-old patients who received collaborative care management for depression.	Patient have access for 12 months to a depression clinical specialist who coordinated depression care with their primary care physician.	Comparison between groups on ‘process of care’ type of treatment and level of care received. Clinical outcomes compared between groups: Symptom checklist (SCL)-20 depression score, treatment response (≥ 50% decrease SCL-20 score).
Boyd, 2009	Cluster RCT	904 of 65 and older and ‘highrisk patients’	To evaluate the effects of “GuidedCare” on patient-reported quality of chronic illness care.	‘Guided care’ integrate a nurse trained in chronic care into a primary care practice to work with 2-5 physicians in providing comprehensive chronic care to 50-60 multi-morbid older patients.	Patient Assessment of Chronic Illiness Care (PACIC)survey by telephone: (Experience of chroniccare) * goal setting, coordinated care, decision support, problem solving, patient activation, aggregate quality
Wu, 2010	RCT	74 long-term care facility resident (aged >70y)	To evaluate the clinical effectiveness of integrated interdisciplinary team care for severely disabled LTCF residents in Taiwan, so to promote better quality of care in this setting.	Integrated care model versus traditional model of care	Physical function, nutritional status, several quality indicators (Quality indicators included unplanned feed tube replacement, unplanned urinary catheter replacement, emergency department visit, hospitalizations, and incidence of urinary infections, pneumonia, and pressure sore.)
Cameron, 2010	SR	Involving 25422 elderly	To present the best evidence for effectiveness of programs designed to reduce the incidence of falls in older people in nursing facilities and hospitals.	Any intervention to reduce falls vs usual care or placebo	Primaryoutcome: ^*^ number of falls ^*^ number of people who fall Secondary outcome: ^*^ severity of falls ^*^ fractures and deaths
Markle-Reid, 2010	RCT	109 elderly 75y and older	This study determined the effects and costs of a multifactorial, interdisciplinary team approach to falls prevention.	Multifactorial, interdisciplinary team approach compared with usual home care services	^*^ number of falls ^*^ fall risk factors (number of slips and trips, functional health status and related quality of lite, nutritional status, gait and balance, depressive symptoms, cognitive function, and confidence in performing ADLS) ^*^ the six-month costs of use health services with a multifactorial, interdisciplinary team approach
Respect team, 2010	Multiple interrupted time-series	551 Aged ≥ 75	To estimate the effectiveness of pharmaceutical care for older people, shared between GPs and community pharmacists in the UK, relative to usual care.	Pharmaceutical care, shared between GPs and community pharmacists in the UK relative to usual care (acted as own control)	Primary outcome: UK Mecication Appropriateness Index (UK-MAI) Secondary outcomes: ^*^ quality of life (SF-36) ^*^ health utility measured by the EQ-5D ^*^ costs of pharmaceutical care ^*^ associated health care to the NHS were also collected
Bryant, 2011	RCT	269 65 years and older on five or more prescribed medicines.	The objective was to determine whether involvement of community pharma-cists undertaking clinical medication reviews, working with general practitioners, improved medicine-related therapeutic outcomes for patients.	Community pharmacists undertook a clinical medication review (Comprehensive Pharmaceutical Care)and met with the patient’s general practitioner to discuss recommendations about possible medicine changes versus usual care.	^*^ Ouality of Life (SF-36) ^*^ Mecication Appropriateness Index.
Ryvicker, 2011	RCT	3290 older chronically ill patients served by a large homecare organization	To describe (1) the impact of a guality improvement initiative (QI) on functional outcomes of older, chronically ill patients served by a large homecare organization; and (2) key implementation challenges affecting intervention outcomes.	A quality improvement initiative on functional outcomes of older, chronically ill patients served by a large homecare organization vs usual care.	Primary outcomes: changes in ADL on patient level (Notes from observations and from semi-structured interviews about how the intervention was implemented during phase 1 and phase 2)
Mudge, 2012	Pre-planned subgroup analysis of controlled trial	1004 aged over 65 and admitted from residential aged care	To identify the impact of an interdisciplinary care model on medical inpatients admitted from residential aged care (RAC).	Interdisciplinary care model on medical inpatients admitted form residential aged care (RAC)	In-hospital mortality for patient from RAC and 6-month mortality compared to patients from the community.
Berglund, 2013	RCT	161 age 80 and older or 65–79 with minimum 1 chronic illness and a need for assistance in ADL	To analyse frail older people’s views of quality of care when receiving a comprehensive continuum of care intervention, compared with those of people receiving the usual care (control group).	Data-collection period: January 2009-October 2011. A comprehensive continuum of care intervention versus usual care. (The intervention included early geriatric assessment case management, interprofessional collaboration, support for relatives and organising of care-planning meetings in older people’s own homes.)	Poeple’s views on quality of care with questionnaire. Scales and items contained: functionl ability, illness, life satisfaction, health, medication and quality of care.
Nazir, 2013	SR	Involving > 33015 elderly	To study the impact of interdisciplinary interventions on health outcomes of NH residents and to document features of successful interventions including those that used formal teams.	RCT’s, NH setting or residential care facilities, team-based interventions and outcomes that were facility or resident based.	(impact on) Resident outcomes as reported in the included studies.
Rantz, 2013	Qualitative research (during randomized two group repeated-measures design)	Nursing homes (72 professionals)	The purpose of this article is to discuss a qualitative analysis of field notes of observational data of the nursing homes that participated in a two-year intervention to improve quality of care, resident outcomes, and organizational working conditions (Rantz et al., 2012). The focus of this analysis was on the use of team and group processes by the nursing home staff in quality improvement efforts.	Facilities in resident outcome “need of improvement” received multilevel intervention designed to help them (quality improvement methods and team and group process for direct-care decision-making…)	The focus of this analysis was on the use of team and group processes by the nursing home staff in quality improvement efforts. Description of behavior of staff in intervention facilities during a RCT for improving quality of care and subsequently improving resident outcomes in nursing homes.

## Results

**Figure 1:**
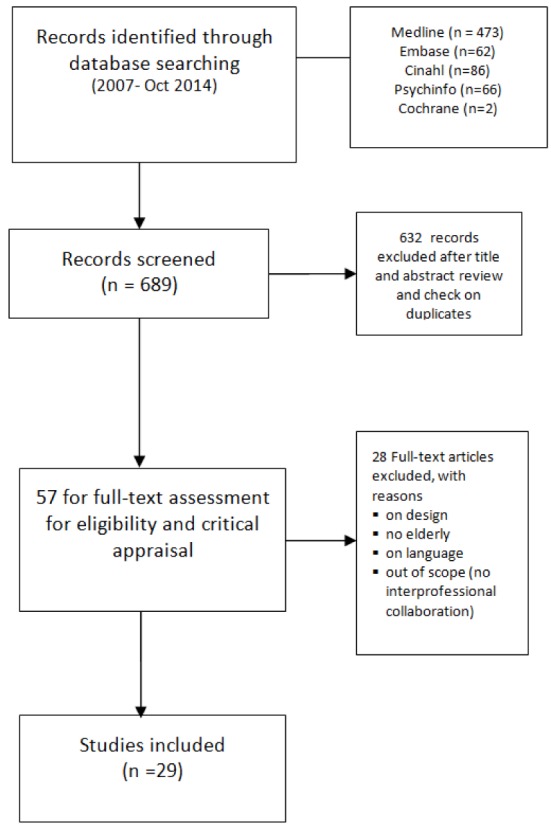
Flowchart results literature search.

Overall, 689 references were identified by the search, of which 57 were eligible on the basis of their title and abstract. Finally, 29 publications met the inclusion criteria after critical appraisal (Table 1) on full text and were included for the review (Figure 1). In general the interventions were described well enough to decide whether an intervention could be identified as ‘interprofessional’ or not. However the description of the control group was not always well described to know the exact difference between ‘interprofessional collaboration’ as intervention and the ‘other’ collaboration.

### Results of the critical appraisal

After the critical appraisal the reviewers labeled the results on outcomes with a category to be able to synthesize the results on outcomes in an overview (Table 3). This strategy brought us to the following categories: ‘Collaboration’ (n = 24), ‘Patient outcome level’(n = 32) and ‘Costs’(n = 4). For nineteen studies of the 29 at least one positive effect including statistical significance was found in advantage of the intervention group and so in favour of interprofessional collaboration. Overall seventeen studies investigated the possible effect of an interprofessional intervention on the category ‘collaboration’, nineteen on ‘patient outcome level’ and four on ‘costs’. The 29 publications included a total of over 80,000 participants and were carried out in 18 different countries.

**Table 3:** Overview of the outcome indicators on interprofessional collaboration.

Reference	Collaboration					Patient level outcomes										Costs

	KE	ID	spr	Spa	QOHC	P	Fl	QOL	1	D	B	T	LOSH	M	PR	

Chapman 2007		NS				S			NS	NS	S					
Counsell 2007					S			S	NS			S				
Denneboom 2007		S														
Stenvall 2007a									S					NS		
Stenvall 2007b							S									
Phelan 2007			NS		NS			NS	NS							
Young 2007				S					S							
Bellantonio 2008												NS				
Bergrenn 2008							NS									
Bonit 2008	S		S		S											
Gates 2008							NS									
Unützer 2008																NS
Boyd 2009	S			S	S											
Counsell 2009																S
Dedhia2009												NS				
Handoll 2009			S						NS				NS	NS	NS	NS
Hogg 2009		S			S											
Stroke Unit 2009								NS	S				S	S		
Van Leeuwen 2009										S						
Cameron 2010							NS									
Markle-Reid2010							S									NS
Respect team 2010		NS						NS								
Wu 2010					NS											
Bryant 2011		S						S								
Ryvicker2011	NS								S							
Mudge 2012	S													S		
Berglund 2013				S												
Nazir 2013	S	S														
Rantz 2013	NS															

KE = Key elements ID = Involved disciplines SPr = Satisfaction professionals Spa = Satisfaction patients QOHC = Quality of Health Care P = Pain FI = Fall incidence QOL = Quality of Life D = Depression I = Independence B = Behaviour T = Transtions LOSH = Length of Stay in Hospital M = Mortality PR = Period of Rehabilitation NS = not significant

### Collaboration

Seventeen out of 24 outcome indicators within the category of ‘collaboration’ reached significant difference in advantage of the intervention group (**Table 3**). Within the category of ‘collaboration’ the sub-indicator outcomes are key elements, involved disciplines, satisfaction by professionals and by patients and finally quality of health care.

### Key elements

Summary key element reported in the studies:

– Goal setting [22,23]

– Team communication [24,25,26]

– Coordination of care decision support [22,23,24,25,26]

– Patient activation [23,25]

– Care (and discharge) planning [24,25,26]

– Kind of contribution of involved disciplines [25,26,27]

– Leadership [25,26,27]

Seventeen of the included studies reached a statistically significant effect of interprofessional collaboration as an intervention by using (organizing) coordinated collaboration or special programs (**Table 3**). Nazir et al (2013) investigated the impact of multidisciplinary rehabilitation on health outcomes of nursing homes residents. Team communication and coordination were confirmed as consistent features for successful collaboration [25]. Mudge (2014) reported in the implementation of an interprofessional care model, including greater allied health staffing, consistent interdisciplinary teams, structured daily interdisciplinary meetings and explicit discharge planning. This interprofessional care model seemed effective for patients admitted from residential aged care [24]. ‘Guided Care’ scored significantly higher on quality of care [22,23]. Participants receiving guided care reported also significant higher scores on knowledge about and satisfaction for goal setting, coordination of care, problem solving, patient activation and aggregated quality in comparison with receivers of usual care, up to 18 months follow up [23]. In the quality improvement initiative in the study of Ryvicker et al (2011), the findings highlight the challenges of relying on peer-to-peer spread, and of distinguishing the core elements of an effective improvement strategy. Leaders should develop explicit communication plans and commit resources to implement the quality improvement initiatives over time [26]. Rantz et al (2013) described the influence of interprofessional teams to sustain quality improvement in nursing homes that ‘need improvement’. Active participation of the leaders increases the chance for success of implementing quality improvement projects [27].

### Involved disciplines

Chapman et al (2007) reported social workers played an important role in coordinating the work of the multidisciplinary team and especially in involving family members in care planning and interventions. Although the teams were significantly effective in reducing agitated behaviour and pain of the residents, no effect was found on the level of collaboration and coordination itself [28]. In two out of three studies [29,30,31] interventions targeting pharmaceutical care including general practitioners and pharmacists showed significant effects. In the study of Denneboom et al (2007) pharmacists suggested the changes in medication to the general practitioners after medication review. Case conferences on prescription-related problems resulted in more medication changes than written feedback [30]. Clinical medication reviews in collaboration with general practitioners can have a significant positive effect on the ‘Medication Appropriateness Index’. However pharmacist withdrawal from the study suggest that community pharmacy may not be an appropriate environment from which to expand clinical medication reviews in primary care [29]. Interviewing patients, development and implementation of pharmaceutical care plans together with patients’ general practitioners and monthly medication reviews with patients performed by pharmacists did not reach any significant changes in appropriateness of prescribing medication [31]. In contrast, participation of primary physicians and/or a pharmacist in the interprofessional intervention, as well as team communication and coordination, were consistent features of successful interventions [25]. It seemed beneficial for the quality of care for chronic disease management to expand traditional family practice with pharmacists or nurse practitioners who focus on the management of this specific group of older, complex patients [32].

### Professional satisfaction

In the study of Boult (2008) guided care had a positive effect on changes in physicians satisfaction for communication with patients, family caregivers, educating family caregivers, motivating patients to participate in maximizing their health, referrals to community resources and change in knowing all the medication patients are taking [22]. The burden of the care in a multidisciplinary rehabilitation for elderly with hip fracture, as rated by the Caregiver Strain Index was reported to be statistically and clinically significantly less for care providers of participants of home-based group [33]. Primary care providers’ satisfaction in the study of Phelan et al (2007) in investigating effective primary care to elderly was positive for intervention but not statistically significant [34].

### Patient satisfaction

When receiving a comprehensive continuum of care intervention, frail older people perceived quality of care significantly higher [35]. More specially the items about care planning in the intervention group were rated higher than the control group at three- and 12 months follow-ups. Guided care also improves self-reported quality of chronic health care for multi-morbid older persons [23]. The reported patient satisfaction for the multidisciplinary team care for elderly was significant higher in community hospitals than in general hospital care [36].

### Quality of health care

In six studies effect on quality of health care was investigated [22,23,32,34,37,38]. In the studies of Boult et al (2008) and Boyd et al (2009) the quality of the health care was measured with the Patient Assessment of Chronic Illness Care (PACIC) [22,23]. In the study of Counsell et al (2007) effect on quality of care was measured with ‘Assessing Care of Vulnerable Elders’ [37]. In the study of Hogg et al (2009) effect on quality of care for chronic disease management was found using a form of collaborative multidisciplinary care teams as intervention [32]. In all four of the above mentioned studies a positive statistical significance was reached in favour for the intervention [32]. In the study of Phelan (2007) and Wu (2010) no statistical difference was found for quality of care indicators [34,38].

### Patient outcome level

On ‘patient outcome level’ only 15 out of 32 outcome parameters were to be understood as effective, by reported statistical significance (**Table 3**). Within the category of ‘Patient level outcome’ the sub-indicator outcomes are pain, fall incidence, quality of life, independence, depression and behavior, transitions, length of stay (LOS) in hospital, mortality and period of rehabilitation.

### Pain

One study found a positive effect of an interprofessional intervention for decreasing pain, using the Faces Legs Activity Cry Consolability (FLACC) and Pain in Advanced Dementia (PAINAD) scales [28].

### Fall incidence

Two studies targeted effects on fall incidence and fall-related injuries and were successful in significantly decreasing fall incidence and slips and trips [39,40]. Three studies, including two systematic reviews, did not report significant decrease of fall incidence as a result of interprofessional interventions [41,42,43].

### Quality of life

Effect on quality of life was found in the study of Counsell et al (2007) implementing a geriatric care management model on improvement of the quality of care [37]. Bryant et al (2011) investigated the influence of involvement of community pharmacists on improvement in medicine related therapeutic outcomes for patients. Quality of life and medication appropriateness index increased because of interdisciplinary pharmaceutical care [29]. There were no statistically significant differences favouring the intervention group in a systematic review on multidisciplinary rehabilitation for elderly with hip fractures [33]. Also in the RESPECT (Randomized Evaluation of Shared Prescribing for Elderly people in the Community over Time) model of wherein pharmaceutical care was shared between community pharmacists and general practitioners, no significantly changes were reported on the quality of life for elderly [31]. Also the Stroke unit study (2009) did not report on statistically significant changes for quality of life [44].

### Independence

In four out of eight studies significant effects were found on independence for older people needing rehabilitation and receiving an interprofessional intervention [26,28,33,34,36,37,40,44].

### Depression and behaviour

The results on clinical outcomes for collaborative care management on treatment response for depression seemed effective on the long-term (24 months) for young-old patients (aged 60–74) [45]. Advanced illness care teams for nursing home residents with advanced dementia were found effective in reducing agitated behaviour and pain but not depression [28].

### Transitions and LOS hospital

In the study of Counsell et al (2007) emergency department visits and hospital utilization were reduced through geriatrics interdisciplinary team that provided ongoing care management [37]. A multidisciplinary team intervention did not significantly reduce the risk of transitions for individuals with dementia relocating to assisted living [46]. Even though hospitalized elderly patients are treated with consideration of their specific needs, health care outcomes visits to emergency departments did decrease, but not significantly [47]. In multidisciplinary rehabilitation participants of the intervention group had overall shorter hospital stays as reported in the systematic review of Handoll [33]. In the study of the stroke unit (2009) for length of stay in in the stroke unit group a modest reduction was found [44].

### Mortality

In four studies [24,33,40,44] mortality was explicitly mentioned, of which in two significant difference was found [24,44]. Stroke patients who received multidisciplinary organized care were more likely to be alive one year after the stroke [44]. Patients admitted from residential aged care receiving the interprofessional intervention had a significant reduction in in-hospital mortality [24].

### Period of rehabilitation

In the study of Handoll (2009) the hospital stay was shorter for the intervention group, but the period of rehabilitation was longer (not statistically) [33].

### Costs

In the category of ‘costs’ only one study reached statistical significance (**Table 3**). In the study of Counsell et al (2009) targeting the costs of interprofessional collaboration programs, neutral cost over two years was reported for patients at high risk of hospitalization from the healthcare delivery system perspective. For patients at low-risk of hospitalization the costs differed statistically significant in disadvantage of the intervention [37]. In three studies with all different periods of measuring costs to use health services with a multifactorial, interdisciplinary team approach, no statistical differences were reported [33,39,48].

## Discussion

The aim of the study was to summarize indicators of effective interprofessional collaboration for elderly. It has to be acknowledged that due to the strict methodology, relevant studies could have been missed. During the process of summarizing the indicators the reviewers categorized the indicators in three categories. This strategy helped to gain insight into what is being investigated in order to measure possible effects of interprofessional interventions. The overall effects of interprofessional interventions are positive, but based on heterogeneous outcomes. Exploring the outcomes gave an overview of outcome indicators with interprofessional collaboration as intervention.

Within the category of ‘collaboration’ the key elements target important criteria for interprofessional collaboration to be measured. Goal setting, team communication, coordination of care decision support, patient activation, care planning and discharge planning, kind of contribution of disciplines and leadership seem to be important key elements for interprofessional collaboration. Moreover, the way of communication and medication appropriateness in pharmaceutical care, seemed important outcome indicators [29,30] that effected the quality of life for patients [29].

Despite the positive effects found favouring interprofessional collaboration on health care outcomes, still too many outcome indicators remain without effect or were reported with a poorness of evidence. Moreover, we noticed that the existing collaboration within the usual care is rarely described. This makes it difficult to fully understand the difference with the usual care and what makes the interprofessional collaboration as intervention effective. From the results it seemed not possible to summarize the process how collaboration was experienced differently from the usual care. From another perspective it is generally accepted that working in an interprofessional team involves group dynamics and leadership. In the systematic review of Nazir et al (2013) this perspective was confirmed [25]. Several studies educated the professionals of the intervention group [22,47,48], but with the information from the publication we could not identify how and with which aim they were trained. It was not clear whether the education was on how to work together or just on being able to perform the intervention as standardized as possible. So no conclusions can be made on learning goals in training to learn to collaborate interprofessionally. In terms of quality of care regarding the definition by Donabedian [49] most of the studies measured effect of interprofessional collaboration on the level of technical performance, only few described the effect on level of interpersonal procedures [22,34,47].

Several outcome indicators concerning interprofessional care effectiveness for elderly on patient level outcome were found. Pain, fall incidence, quality of life, independence for daily life activities, depression and agitated behaviour, transitions, length of stay in hospital, mortality and period of rehabilitation seem the most prominent outcomes in the included literature to identify effect of interprofessional collaboration for this specific population. However, as mentioned in the study of Rantz (2013) [27], teams can fully, partial or not adopt new ways of working when implementing interprofessional collaboration strategies. This should always be taken into account when teaching and so implementing models of interprofessional collaboration in practice. If one wants to show effect of interprofessional collaboration, the intervention should also last long enough and be well described so difference with usual care is also clear.

To enhance insights in possible bottlenecks in interprofessional care delivery it can be important to include the influence of professional and personal relationships within the team and with the patients. In the studies of Nazir (2013) [25] and Boult (2008) [22], the professional relationships as key elements were very well described. This gave insight in how interprofessional collaboration is to be understood in their context. Also the patients appreciated the knowledge about the goals of the care they received. Therefore it seems important that interprofessional collaboration is to be clearly described and implemented long enough to know what effects it can have on patient level. Based on the three included studies involving costs of interprofessional collaboration, no general conclusion can be drawn on that category.

## Conclusion

Overall, outcome indicators of interprofessional collaboration for elderly with a significant effect can be summarized in three main categories: ‘collaboration’, ‘patient level’ and ‘costs’. For ‘collaboration’ the outcome indicators for IPC are key elements of collaboration, involved disciplines, professional and patient satisfaction and quality of care. On ‘patient level’ the outcome indicators are pain, fall incidence, quality of life, independence for daily life activities, depression and agitated behaviour, transitions, length of stay in hospital, mortality and period of rehabilitation. ‘Costs’ of interprofessional interventions on short-and long-term for elderly need further investigation. When organizing interprofessional collaboration or interprofessional education these outcome indicators can be considered as important topics to be addressed. Overall more research is needed to gain insight in the process of interprofessional collaboration and so to learn to work interprofessionally.

## Competing Interests

The authors declare that they have no competing interests.

## Annex: Search Strings

1. exp Interprofessional Relations/ and (collaborat$ or team$).tw.
2. exp Patient Care Team/ and (collaborat$ or team$).tw.
3. ((interprofessional$ or inter-professional$) adj (collaborat$ or team$)).tw.
4. ((interdisciplin$ or inter-disciplin$) adj (collaborat$ or team$)).tw.
5. ((interoccupation$ or inter-occupation$) adj (collaborat$ or team$)).tw.
6. ((multiprofession$ or multi-profession$) adj (collaborat$ or team$)).tw.
7. ((multidisciplin$ or multi-disciplin$) adj (collaborat$ or team$)).tw.
8. ((multioccupation$ or multi-occupation$) adj (collorat$ or team$)).tw.
9. ((transdisciplin$ or trans-disciplin$) adj (collaborat$ or team$)).tw.
10. (team$ adj collaborat$).tw.
11. or/1–10
12. randomized controlled trial.pt.
13. controlled clinical trial.pt.
14. randomized controlled trials/
15. random allocation/
16. double blind method/
17. single blind method/
18. or/12–17
19. animals/not humans/
20. 18 not 19
21. 11 and 20
